# Genion, an accurate tool to detect gene fusion from long transcriptomics reads

**DOI:** 10.1186/s12864-022-08339-5

**Published:** 2022-02-14

**Authors:** Fatih Karaoglanoglu, Cedric Chauve, Faraz Hach

**Affiliations:** 1 School of Computing Science, Simon Fraser University, Burnaby, BC Canada; 2 Vancouver Prostate Centre, Vancouver, BC Canada; 3 Department of Mathematics, Simon Fraser University, Burnaby, BC Canada; 4 Department of Urologic Sciences, The University of British Columbia, Vancouver, BC Canada

**Keywords:** Gene fusion detection, Long-read sequencing, Transcriptomics, Dynamic programming

## Abstract

**Background:**

The advent of next-generation sequencing technologies empowered a wide variety of transcriptomics studies. A widely studied topic is gene fusion which is observed in many cancer types and suspected of having oncogenic properties. Gene fusions are the result of structural genomic events that bring two genes closely located and result in a fused transcript. This is different from fusion transcripts created during or after the transcription process. These chimeric transcripts are also known as read-through and trans-splicing transcripts. Gene fusion discovery with short reads is a well-studied problem, and many methods have been developed. But the sensitivity of these methods is limited by the technology, especially the short read length. Advances in long-read sequencing technologies allow the generation of long transcriptomics reads at a low cost. Transcriptomic long-read sequencing presents unique opportunities to overcome the shortcomings of short-read technologies for gene fusion detection while introducing new challenges.

**Results:**

We present Genion, a sensitive and fast gene fusion detection method that can also detect read-through events. We compare Genion against a recently introduced long-read gene fusion discovery method, LongGF, both on simulated and real datasets. On simulated data, Genion accurately identifies the gene fusions and its clustering accuracy for detecting fusion reads is better than LongGF. Furthermore, our results on the breast cancer cell line MCF-7 show that Genion correctly identifies all the experimentally validated gene fusions.

**Conclusions:**

Genion is an accurate gene fusion caller. Genion is implemented in C++ and is available at https://github.com/vpc-ccg/genion.

## Background

Gene fusions are aberrations that result from genomic events such as deletions, inversions or translocations, whereby segments of two genes become closely located and are transcribed together into a *chimeric RNA* molecule. Gene fusions may hinder the original functions of the fused genes and can introduce functional novelty [1]. We refer to the study by Wu et al. [2] for a detailed discussion on gene fusions and chimeric RNAs.

Gene fusions are observed in many types of cancer. For example, the *BCR-ABL1* fusion, caused by a balanced reciprocal translocation between chromosomes 9 and 22, is observed in chronic myeloid leukemia [3]. This gene fusion causes genome instability and impairs signaling pathways [4]. Gene fusions between androgen regulated genes and genes from the ETS family are observed in many prostate cancer patients [5, 6]. TMPRSS2-ERG is one of such fusions created by the deletion of ∼2.8Mb between these genes. This fusion is observed in ∼50*%* of prostate cancer patients and is associated with high expression of estrogen regulated gene (ERG) [7]. Several studies suggest that recurrent gene fusions can be used as potential biomarkers in cancer [8, 9]. Moreover, gene fusions are not specific to cancer and are also important for other diseases [10–12]. All this underscores the importance of detecting accurately gene fusions.

Traditionally gene fusion discovery is done using short reads generated by Illumina sequencing. Gene fusion discovery with short paired-end reads is a well-studied problem and many tools have been developed to address it. These tools use approaches based on either reads mapping or assembly. We refer to Haas et al. [13] for a recent review focusing on gene fusions in cancer, showing that mapping-based methods generally outperform assembly-based methods. Detecting gene fusion from RNA-seq short reads is a very active research area, with novel methods being published regularly (e.g. Chiu et al. [14], Dehghannasiri et al. [15]).

While RNA-seq short reads are adequate for calculating gene expression, they do not perform as well for the discovery of gene fusions caused by genomic events. This is mainly due to the weakness of the signal provided by mapped short reads, especially in the case of splicing events that results in split mappings of the reads around gene fusion breakpoints. Furthermore, gene fusions are not the only possible cause resulting in chimeric RNA reads; *read-through* and *trans-splicing* are biological events that generate chimeric RNA molecules during or after transcription [16, 17]. Additionally, library preparation and sequencing can lead to the creation of artifactual chimeric RNA reads. It is generally difficult to differentiate chimeric RNA reads caused by sequencing or non-genomic transcriptional events from actual gene fusion reads that are caused by genomic events without using complementary genome sequencing data. This issue has important implications toward the detection of potential cancer biomarkers.

Transcriptomics Long-Read Sequencing (TLRS) is an emerging technology that offers an opportunity to overcome these issues [18]. TLRS is a promising approach for the low-cost detection of gene fusions while allowing the precise characterization of the fusion isoforms. To the best of our knowledge, AERON [19] (still in development) and LongGF are the only published tools that are capable of detecting gene fusions from TLRS. AERON uses a sequence to graph alignment approach to find gene fusions, while LongGF uses splice aware mappings to call gene fusions.

While at first, it may seem that gene fusion discovery from transcriptomics long reads might not be difficult due to the length of the reads that can span the full length or large parts of the transcripts, we show that the various mechanisms creating chimeric RNA reads introduce non-trivial challenges. While finding the reads that map over multiple genes, indicating potential gene fusions, is straightforward, the challenging part of gene fusion discovery is to identify such reads supporting true fusions, which form a minority of the multi-gene reads. For example, in our experiments on PacBio IsoSeq data for the MCF-7 cell line [20], out of ∼2.4 million reads, ∼180,000 reads were mapped to multiple genes, but only ∼2,000 supported high confidence gene fusions. To address these challenges, we introduce a new computational gene fusion discovery method, Genion (**GEN**e fus**ION**). From the mapping of transcriptomic long reads to a reference genome, Genion first identifies chains of exons. Reads with chains that contain exons from several genes provide an initial set of reads supporting potential gene fusions. Then, Genion clusters the reads that indicate potential gene fusions to define fusion candidates and ranks these fusion candidates, using a statistical approach based on the analysis of background expression patterns of the normal transcripts for the involved genes and on the co-occurrence of the fusion candidates in other potential fusion events.

In order to evaluate the sensitivity and accuracy of Genion, we generated simulated data by spiking known gene fusions from the Cosmic database [21] into a human transcriptome with an expression profile defined from data from the 22Rv1 prostate cancer cell line. On this simulated data, Genion performed comparably to LongGF, the only other long reads fusion discovery tool we could run on this data, by identifying one more gene fusion. Furthermore, we evaluated Genion on a breast cancer cell line, MCF-7, where both Genion and LongGF identified three experimentally validated fusions successfully. As for this dataset, for 13 additional fusions, validated from short reads, although LongGF did find 5 more gene fusions than Genion, we suspect that some of the reported gene fusions were in fact incorrect due to features suggesting they might be false positives. Moreover, the total number of the fusions calls reported by LongGF is 9× the number of fusions called by Genion and ∼40*%* of the LongGF fusion calls were shown to be random pairing by Genion. Finally, we compared the two tools on an in-house dataset generated from the prostate cancer cell line 22Rv1 [22], a well-studied cell line known for expressing different variants of the Androgen Receptor (AR) gene. We are not aware of any validated fusion on this cell line and we believe that this cell line can play a unique role as a negative control. On this dataset, Genion reported only one gene fusion while LongGF reported 70 gene fusions, including the one reported by Genion.

## Implementation

### Preliminaries

**Terminology** As the terminology for gene fusions is inconsistent between different studies, we first formally define the terminology that will be used throughout this manuscript. *Chimera* or *chimeric read* refers to any RNA read that contains sequences from multiple genes. To denote a chimera of genes *A* and *B*, we use *A*:*B*. Chimeras can be classified into five categories: (i) *Gene fusion*: a chimera created by a genomic structural variation such as deletion, inversion or translocation [23]. (ii) *Transcriptional read-through*: a read sequenced from a molecule that is formed by the splicing of exons of multiple genes that are on the same strand and are in close proximity of each other [17]. Read-through events are caused by non-genomic events and tend to join the exons of the 5^′^ gene to the exons of the 3^′^ gene [17]. Recurrent read-through transcripts have been observed in breast and prostate cancers [17, 24] and may have biological implications. (iii) *Trans-splicing*: a form of RNA processing whereby exons from two different primary RNA transcripts are ligated [16]. (iv) *Library preparation random-pairing*: a common artifact where different RNA molecules randomly attach together during the library preparation (e.g. due to template switching [25]). (v) *Base calling random-pairing*: a chimera that is caused by erroneous segmentation by the base calling software which result in two molecules being represented by a single read. This artifact is specific to Nanopore sequencing and not observed in PacBio data.

**Fig. 1:**
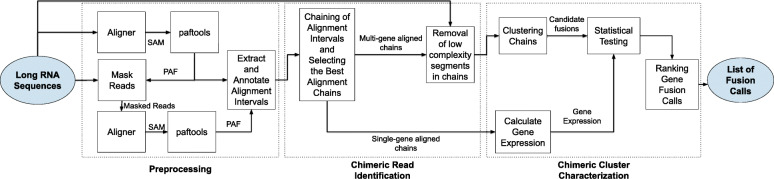
Genion pipeline. **Preprocessing** step produces alignment intervals for each input read. Paftools [30] is used to convert the mappings from SAM to PAF format. Mapped parts of the reads are masked and mapped again. Outputs of mapping steps are merged and converted to set of segments and annotated. **Chimeric Read Identification** step chains the annotated segments of each read and identifies the gene content. **Chimeric Cluster Characterization** step takes single-gene aligned chains to calculate gene expressions. Multi-gene aligned chains are clustered. Each cluster is statistically tested and FDR correction is applied on calculated *p*-values. For each cluster FiN and ff-igf scores are calculated. Clusters are characterized and ranked according to these scores and reported

**Overview** Genion starts by mapping reads to the human reference genome. These mappings are then processed to extract sets of interval pairs, referred as *segments*, representing the positions of mapped regions on the reads and on the reference genome. These segments are matched with annotated exons that overlap with them. Using a dynamic programming exon chaining algorithm, Genion associates each read with one or more sets of exons that are expressed in the read. We call each such set an *exon chain*. These chains are clustered into groups of chimeric reads that involve the same two different genes. Each such cluster is statistically tested to remove candidates likely originating from random pairing. Then, each cluster is analyzed during a post-processing step in order to define its likely origin (gene fusion, read-through or trans-splicing). Overall, Genion has three main stages: (i) pre-processing, (ii) chimera identification, and (iii) gene fusion calling. Figure 1 depicts an overview of the Genion pipeline which we describe in detail in the following sections.

### Pre-processing

In this stage, Genion maps the TLRS data onto the human reference genome and for each read, it produces a list of pairs of aligned intervals, one on the read and one on the genome, together with an annotation of the read in terms of exons contained in these intervals.

**Mapping transcriptomics reads.** We first map all TLRS reads to a reference genome (human genome version GRCh38) using the splice-aware mapper deSALT [26]. We mask the mapped parts of the reads and map the masked reads using again deSALT with the same settings to find mappings from the parts of the reads that were initially unmapped and merge the two sets of mappings. We then associate to each read a set of mapped *segments*, composed each of a read interval and the corresponding genome interval it maps to. Note that read intervals do not overlap while genome intervals might overlap. In order to account for potential indels (due to sequencing errors [27]) that would disrupt such segments, Genion merges consecutive segments if the corresponding intervals are less than 10 nucleotides apart both on the read and on the reference genome.

**Annotating segments.** Next, we annotate each segment with the set of exons its genome interval overlaps with. For this task, we use the Ensembl human gene annotation (release 97) database [28]. This database contains 60,617 genes (including coding and non-coding), known isoforms of these genes and exons present in each isoform. We build an *interval tree* of the annotated exons, a data structure that allows Genion to detect quickly and accurately all the overlaps between exons and genome intervals, with no restriction on the length of the overlap.

Note that more than one exon can annotate each segment as adjacent exons may be close enough to overlap with a single segment, different isoforms of the same gene may have overlapping exons or exonic regions of two distinct genes may overlap. Furthermore, if the set of the exons associated with a segment is empty, this likely implies it belongs to an intronic or an intergenic region.

The result of this preprocessing phase is the annotation of each segment resulting from the mapping by a set of exons, that will be used as features associated to reads in the next phase aimed at detecting potentially chimeric reads.

### Chimeric reads identification

In this step we aim to associate to each read an ordered list of *non-overlapping unique* exons, that we call an *exon chain*. Exon chains will be used as a coarse encoding of reads in order to cluster reads into groups likely originating from the same isoform. Such clusters will then be used in two ways: (1) clusters associated to multi-gene exon chains will be considered as a ground set that contains reads from potential gene fusion isoforms, and (ii) clusters associated to single-gene exon chains will be used to calculate the expected expression of individual genes, an important feature to filter out false positive candidate gene fusions in a later step.

**Obtaining optimal exon chains.** To compute exon chains, we devised an algorithm inspired by co-linear chaining dynamic programming (DP) algorithms (see [29] for a recent reference on chaining algorithms).

Our algorithm aims to maximize the exonic sequence content of a chain while minimizing the number of gene/isoform switches between two consecutive segments.

In order to explain the algorithm, we define the notations that are used in the DP equations. A given read *r* has a set of segments. Each segment is a triplet (*I*_*r*_,*I*_*G*_,*D*) where *I*_*r*_=(*r*_*s*_,*r*_*e*_) is a read interval, *I*_*G*_=(*g*_*s*_,*g*_*e*_) is a genome interval, and *D*={−1,+1} is the direction of the mapping of *I*_*r*_ to *I*_*G*_. Each *I*_*G*_ overlaps with at least one isoform, and each overlap is represented by a triplet (*I*_*e*_,*GI*_*e*_,*TI*_*e*_) where *I*_*e*_=(*g*_*es*_,*g*_*ee*_) is an isoform interval, *GI*_*e*_ is a gene identifier and *TI*_*e*_ is an isoform identifier. Thus, each read can be represented by a list *M*=[*m*_1_,*m*_2_,…,*m*_*n*_] of 6-tuple $m_{j}=(I^{j}_{r}, I^{j}_{G}, D^{j}, I^{j}_{e}, GI^{j}_{e}, TI^{j}_{e})$. This set represents the association between segments and exons.

Genion performs the DP per read independent of the other reads and computes for each read a list *S* where *S*[*c*] is the maximum score of chains ending with *M*[*c*]. First, we calculate the overlap between the genomic interval of every segment and its overlapping exons through the reciprocal overlap formula given in (Eq. 1), which is defined as the size of the intersection divided by the size of the larger interval. Second, we find all the parents of *c* through Eq. 2: *p* is a parent of *c* if the read interval of *p* precedes the read interval of *c* and their respective genomic intervals are preceding each other in the direction of the mapping (e.g. if on the forward strand, $I_{G}^{p}$ occurs before $I_{G}^{c}$). Note that if the direction of the mapping of *p* and *c* is different, there is no restriction on the order of their genomic intervals. This is done to consider the chaining of transcripts from fusions caused by genomic events such as inversions or translocations that may fuse two genes on opposite strands. Finally, *S*[*c*] is calculated by iterating over all the parents of *c* and finding the best score through Eq. 3. Note that we use a different penalty value to penalize gene/isoform switching. Thus, our algorithm prioritizes exon chains that are coming from a single isoform over a single gene (isoform switching), and chains from single gene over gene switching (potential fusions). Gene switching is penalized to prefer normal gene chains over fusion chains if their scores are similar. Penalizing the isoform switching does not change the final result. It is implemented to clean up the resulting chain by avoiding alternating exons from different isoforms.

1 $$  \text{overlap}(a,b) = \max\left(0,\frac{\min(a_{e},b_{e}) - \max(a_{s},b_{s})}{\max(a_{e}-a_{s},b_{e}-b_{s})}\right)  $$

2 $$ {}\text{parents}(c) = \left\{ p\ \begin{array}{|l} \ p \in M,~\\[.5ex] (I^{p}_{r_{e}} < I^{c}_{r_{s}}) \land \\[.5ex] \left(D^{p} = D^{c} \land \left(D^{p} \times (I^{c}_{G_{e}} - I^{p}_{G_{s}}) > 0 \right)\right) \end{array} \right\}  $$

3 $$ \begin{aligned} S[c] &\leftarrow \max_{p \in \text{parents}(c)} \{ (S[p]) + \text{overlap}(I_{G}^{c},I_{e}^{c})\\ &\quad\times \text{length}(I_{G}^{c}) \times \text{penalty}(c,p)\} \end{aligned}  $$

4 $$  \text{penalty}(c,p) = \left\{\begin{array}{ll} 1, & \text{if}\ TI_{e}^{c} = TI_{e}^{p}\\ 0.9, & \text{if}\ GI_{e}^{c} = GI_{e}^{p} \land TI_{e}^{c} \neq TI_{e}^{p} \\ 0.5, & \text{if}\ GI_{e}^{c} \neq GI_{e}^{p} \end{array}\right.  $$

The optimal chaining score is given by

5 $$ B = \underset{\,1 \leq i \leq n}{\text{argmax}} \left(S[i]\right),  $$

and to specify an optimal exon chain, we backtrack from the index *S*[*B*] and at each iteration we report (*I*_*e*_,*GI*_*e*_,*TI*_*e*_).

**Preliminary Classification of the exon chains.** After this chaining step, Genion classifies input reads into three categories: (i) if the chain contains intervals from multiple genes, the read is classified as a chimeric read, (ii) if there is only one gene in the chain, it is a normal RNA read, (iii) if the algorithm returns no chain, then it is an intergenic read. Chimeric reads form the ground set from which fusion candidates are obtained.

**Removal of ambiguous chimeric chains.** In this step we filter out chains due to overlapping genes, homologous genes and segmental duplications.

First, if any overlap exists between the genes in the chain, the chain is discarded.

Some of the chimeric reads result from ambiguous mappings where different parts of the same read maps to homologous genes – where two genes shares a high sequence similarity. If the exon chain of a read contains gene partners from homologous gene pairs, we filter them as they may increase the false positive rate in later stages. In order implement this filter, we build a simple index of homologous gene pairs, by mapping the human transcriptome reference to itself using minimap2 [30], where any two genes that are aligned are added to this index.

Segmental Duplications (SD), also known as low-copy repeats, are genomic segments larger than 1 Kb that are duplicated one or more times in a given genome with a high level of homology [31]. These SDs can lead to increased mapping ambiguity since they may involve partial gene duplication in another gene that will not be included in our homologous gene pairs index. To remove likely mapping artifacts due to SDs, we filter out the chimeric chains that are in SD regions. We use GenomicSuperDups [31, 32] that records interval pairs on the human genome where segmental duplications exist. We use again an interval tree data structure to build an interval to interval index of SDs. For each chimeric chain, we search for the range of the first gene using the SD interval tree. If any SD involving the corresponding interval is found, we search for an interval that overlaps with the second gene. If an SD exists between the genes, the chain is discarded.

**Removal of low complexity exon chains.** Our chaining algorithm does not consider the sequence content of the reads and it may chain the poly-A regions of the reads if they are not removed from raw reads as they are mapped by the deSALT. To handle this issue, we remove segments in the output chains if the sequence content consists of more than 70% of a single base.

### Chimeric reads characterization

Given the chimeric read chains and normal read chains, Genion aims to categorize the chimeric chains into three classes: (i) random-pairings, (ii) read-throughs, and (iii) gene fusions candidates. Note that, in the presence of random-pairings created by template switching and segmentation errors, it is difficult to argue a chimeric candidate originates from trans-splicing unless it has significant expression. In this study, we are not targeting trans-splicing chimeras. Trans-splicing with low expression are reported as random-pairing and trans-splicing with high expression are reported as gene fusions. To achieve this goal, Genion estimates the gene expression of each gene from normal read chains, and cluster the chimeric chains into groups of gene pairs. Finally, it performs two tests to differentiate between the three classes introduced above.

**Gene Expression Estimation.** The single-gene chains are used to estimate the gene expression of the genes observed in the sample. For each gene, Genion counts the number of the single-gene exon chains for this gene and stores this value for further characterization of the chimeric clusters. We will refer to the expression of gene *A* as *E*_*A*_.

**Clustering of the chimeric chains.** Chimeric chains are clustered according to the involved gene pairs and we denote the number of supporting chimeric chains for each pair of genes *A* and *B* by *N*_*A*:*B*_. Any chimeric cluster that is supported by less than 3 chimeric chains (user adjustable parameter) is marked as a low support cluster and is discarded.

**Classification of chimeric clusters.** Finally, Genion assigns each chimeric cluster to one of the three following classes: (i) random pairing, (ii) read-through, and (iii) gene fusion. Genion calculates two scores named **F**usion inverse **N**ormal (FiN) and **f**usion **f**requency **i**nverse **g**ene **f**requency score (ff-igf). These two scores differentiate between different type of candidates and order the candidates in terms of Genion’s confidence as to their correctness, as explained in the next paragraph.

**Statistical testing of gene fusion candidates.** To differentiate gene fusions from random pairings, we introduce a simple statistical model. Let *A* and *B* be two unfused genes and *A*:*B* the chimera containing both *A* and *B*. We expect to sequence *mean*(*E*_*A*_,*E*_*B*_)∗*p*_*rp*_*A*:*B* chimeras between these genes, where *p*_*rp*_ is the probability of random pairing occurring during sequencing. For each chimeric candidate, we test the null hypothesis that *N*_*A*:*B*_∼*mean*(*E*_*A*_,*E*_*B*_)∗*p*_*rp*_ indicating that number of chimeric reads is not significantly more than what we would expect from random pairing. We test this null hypothesis using the one-tailed version of Fisher’s exact test as we are not interested in candidates that have significantly less support than expected from a random-pairing. We model the number of random pairings with a hypergeometric distribution that models number of successes without replacement. If we observe *E*_*A*_ and *E*_*B*_ normal reads of genes *A* and *B*, the probability of observing *N*_*A*:*B*_ chimeric reads will be

$$Pr(x=N_{A:B})=\frac{{n \choose N_{A:B}}{n \choose n * p_{rp}}}{{n \choose n * p_{rp} + N_{A:B}}}, $$

where *n*=*mean*(*E*_*A*_,*E*_*B*_)+*N*_*A*:*B*_ and *x* is the random variable recording the number of observed chimeric reads.

The *p*-value for a one-tailed Fisher’s exact test is the probability of observing at least *N*_*A*:*B*_ chimeric reads *Pr*(*N*_*A*:*B*_≤*x*) (Fig. 3). We use false discovery rate (FDR) control on the *p*-values computed using the Fisher’s exact test. We decided to use Benjamini-Yekutieli [33] procedure due to the dependency between the candidates (caused by shared member genes and global random pairing rate used to calculate expected number of random pairings). This procedure reports the corrected *p*-value for each chimeric candidate and reports if it rejects the null hypothesis. This ensures the precision to be (1 - FDR); note that this is the precision of differentiating random pairings from gene fusions, not the final precision of the gene fusions called by Genion.

*FiN Score.* For a chimeric cluster between genes *A* and *B*, the FiN score (FiN_*A*:*B*_) is defined as the ratio of the number of fusion supporting reads to the sum of the numbers of non-fusion supporting reads plus 1.

6 $$  \text{FiN}_{A:B} = \frac{N_{A:B}}{1+E_{A}+E_{B}}  $$

**Fig. 2:**
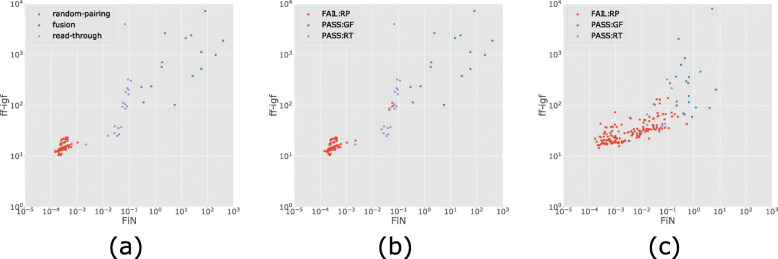
FiN and ff-igf values for each call made by Genion. **a** Ground truth of the simulated dataset, **b** Genion calls on the simulated dataset and **c** Genion calls on the MCF-7 Pacbio dataset. Simulated gene fusions, read-throughs and random-pairings are colored blue, purple and red respectively. Calls are colored with simulated ground truth in (**a**) and Genion predictions in (**b**) and (**c**) [44]. PASS:GF, PASS:RT and FAIL:RP represent chimeras called as gene fusions, read-throughs and random-pairings in (**b**) and (**c**). Chimeras filtered due to overlaps, homology or low support are not included in this figure

In our experiments we observed (Fig. 2) the following relation of FiN score between different types of chimeras:

$${}0 \sim \text{FiN}_{\text{Random Pairing}} < \text{FiN}_{\text{Read Through}} < \text{FiN}_{\text{Gene Fusion}} $$

Intuitively, the FiN score for random pairings is expected to be very small, since it is unlikely that the same random pairing occurs more than once. The FiN score for read-through candidates is still expected to be small but larger than for a random pairings since the likelihood of a read-through transcription occurring more than once is greater. Finally, the FiN scores of true gene fusions due to genomic events are expected to be higher than for read-through candidates, as individual genes involved in a read-through are also transcribed individually, unlike for gene fusions. This value will be close to the fusion read count for homozygous events and it will be reduced by incomplete sequencing of the transcripts and non-fusion reads for heterozygous events.

*ff-igf Score.* We use the ff-igf score to rank the computed chimeric clusters. This score is based on the fact that a gene can be involved in at most two different gene fusions (once on each copy of a diploid genome). We expect any gene to be at most part of a single gene fusion event. Thus, observing a gene in multiple chimeric clusters lowers the confidence it is actually a *bona-fide* gene fusion cluster. The fusion frequency inverse gene frequency score (ff-igf) for a chimeric cluster is the ratio of the number of chimeric chains supporting the fusion *A*:*B* to the sum of the number of chimeric chains from other chimeric clusters containing the gene *A* or the gene *B*. If we denote by *F* the set of all discovered chimeric clusters, it is defined as follows:

7 $$ \begin{aligned} \text{ff-igf}_{A:B} = \log\left(1+ | PG_{A} \cap PG_{B}|\right) \times \log\left(\frac{|F|}{1+|PG_{A}|+|PG_{B}|}\right)\\ \end{aligned}  $$

$$PG_{G} = \left\{X\ |\ N_{X:G} > 1 \lor N_{G:X} > 1\right\} $$

Genion first identifies the read-through chimeras from all candidates. This is because expression of read-through chimeras cannot be estimated and they cannot be differentiated from random-pairings with statistical testing. Chimeric candidates are reported as read-through if:

- Member genes are on the same chromosome, they are on the same strand and distance between the genes is less than 500Kb.
- The FiN score is less than 0.5
- The ratio of the exon chains having second to last exon of the head gene (first gene in the gene pair) and second exon of the tail gene (second gene in the gene pair) over all chains in the cluster is greater than 0.8.

Remaining chimeric clusters are identified as gene fusion or random-pairing depending on rejection of the null hypothesis with the Benjamini-Yekutieli procedure.

Finally, gene fusions candidates are sorted based on their ff-igf score and reported as a ranked list of gene fusion calls.

## Results

LRTS is a relatively new technology. Hence the number of publicly available datasets is limited compared to short reads datasets. In this paper we used both in-house data consisting of nanopore cDNA sequencing of the 22Rv1 prostate cancer cell line, and publicly available nanopore cDNA sequencing data of Universal Human Reference (UHR) and PacBio IsoSeq sequencing data of the MCF-7 breast cancer cell line. We also tested Genion on simulated data.

### Simulated data

**Data generation.** To accurately test the performances of gene fusion discovery tools, simulations need to account for the expected noise and artifacts of real datasets: they should have a realistic expression of regular and fusion genes and should simulate non-genomic events resulting in chimeric reads such as read-throughs and random pairings.

In order to generate data with a realistic level of gene expression, we mapped our in-house nanopore MinION sequencing data of the 22Rv1 prostate cancer cell line to the ENSEMBL 97 human cDNA reference using minimap2 and used the primary mappings to estimate an expression profile for each gene.

To define ground truth gene fusions, we selected 16 validated gene fusions from the most common fusions listed in the Cosmic database [21]. We refer to member genes of these fusions as head and tail genes depending to the order of transcription. In this simulation, we assume that fusions are expressed similar to their head genes. We simulated heterozygous fusions with an expression equal to half of the expression of the head gene. Similarly, we simulated homozygous fusions with an expression equal to the expression of the head gene. We modified the expression of the member genes of each gene fusion to 50% for heterozygous fusions and 0 for homozygous fusions. Out of 16 simulated gene fusions, 10 were homozygous and 6 were heterozygous.

To simulate artifacts resulting in non-fusion chimeric reads, we also added 37 read-through events to the simulation, selected from recurring read-through events [34]; 17 of the read-through events had less than 3 reads in the simulation and were not considered in the performance evaluation. Following published evidence [35] on the exon structure of read-throughs, we simulated transcripts containing all of the exons except the last exon of the head gene and the first exon of the tail gene. Simulated read-through transcripts were assigned an expression level equal to 5% of the head gene expression.

We used the BadRead simulation tool [36] to generate long reads with the calculated expression profile and the ONT MinION error profile. In this simulation, we generated 2,926,804 reads, where 2,900 of these came from the 16 gene fusions. We only simulated reads from chromosomes that contain gene fusions.

Finally, to include in our simulations artifactual chimeric reads due to random pairing, we randomly selected 1% of the simulated reads and randomly paired them into chimeric pairs, resulting in 28,971 randomly paired chimeras, 74 of which originating from a gene fusion or a read-through.

**Tools configuration.** We compared Genion against the two available long-read specific gene fusions discovery tools: LongGF and AERON. AERON is an unpublished method and failed to run on all the datasets we considered in this paper and thus we removed it from our comparisons^1^. For the LongGF tool we followed the instructions in the code repository and mapped the simulated reads using minimap with the “-x splice:hq” option, sorted the reads by name, and ran LongGF with the options (“100 50 100 0 0 2 64”). We ran Genion with default options, which uses the deSALT mapper with parameter “-N 10”, which limits number of reported mappings for each reads.

**Evaluation of chimeric read clustering.** To compare the accuracy of both tools in terms of clustering chimeric reads, we used the classical Adjusted Rand Index (ARI) statistics [37]. The ARI measures the similarity of two clustering, one of them being a ground truth clustering.

True positive (TP), true negative (TN), false positive (FP) and false negative (FN) numbers for ARI calculation defined as follows: TP is the number of read pairs (*r*_1_,*r*_2_) that are in the same cluster in the ground truth as well as in the predicted cluster. TN is the number of read pairs that are in different clusters in the ground truth and the predicted clusters. FP is the number of read pairs that are in the same predicted cluster but in different clusters in the ground truth. FN is the number of read pairs that are in different predicted clusters but in the same cluster in the ground truth. In the context of the fusion calling, FN includes the reads missed by fusion calling method while FP includes random pairings between the same gene pair of an existing gene fusion for both tools. We did not include such cases in the ARI computation. We used the scikit-learn [38] package to compute the ARI.

We also tested Genion clustering accuracy on chimeric reads, not limited to gene fusions. Out of 33,217 chimeric reads originating from a true gene fusion, a read-through or a random pairing, Genion correctly clustered 28,640 of them, giving an ARI of 0.925. For each tool, we labelled the 2,900 simulated fusion reads with the fusion cluster they belong to. If a read is not clustered in a fusion cluster or put into a cluster calling a gene fusion different from its label, it is considered as a singleton cluster for the purpose of computing the ARI. Genion and LongGF clustered respectively 2,251 and 872 fusion reads, giving ARI scores 0.910 and 0.183 respectively (Table 1) when considering only fusion read clusters.

**Table 1:** Clustering statistics for Genion and LongGF and number of fusion reads recovered by each tool

	Reads	Fusion ARI	All ARI
Genion	2,521	0.901	0.925
LongGF	872	0.183	NA

**Evaluation of the fusion calling accuracy.** Genion and LongGF found respectively 15 and 14 of the 16 simulated fusions (Table 2). Genion was the only tool to find the TMPRSS2:ERG fusion. Both tools failed to identify the NAB2:STAT6 fusion. Genion successfully identified the chimeric chains for NAB2:STAT6, however since these two genes are overlapping, the chimeric chains were filtered out. For LongGF, we suspect that NAB2:STAT6 is filtered out for the same reason as Genion, and that TMPRSS2:ERG is not reported because the length of the head gene involved in the fusion is only 80b.

**Table 2:** Simulated gene fusions called by Genion and LongGF

Gene1	Gene2	Genion	LongGF	Gene1	Gene2	Genion	LongGF
TCF3	PBX1	$\checkmark $	$\checkmark $	KMT2A	MLLT3	$\checkmark $	$\checkmark $
JAZF1	SUZ12	$\checkmark $	$\checkmark $	BCR	ABL1	$\checkmark $	$\checkmark $
DNAJB1	PRKACA	$\checkmark $	$\checkmark $	TMPRSS2	ERG	$\checkmark $	✗
KIAA1549	BRAF	$\checkmark $	$\checkmark $	CCDC6	RET	$\checkmark $	$\checkmark $
NAB2	STAT6	✗	✗	CBFA2T3	GLIS2	$\checkmark $	$\checkmark $
EWSR1	FLI1	$\checkmark $	$\checkmark $	PML	RARA	$\checkmark $	$\checkmark $
SS18	SSX1	$\checkmark $	$\checkmark $	RUNX1	RUNX1T1	$\checkmark $	$\checkmark $
COL1A1	PDGFB	$\checkmark $	$\checkmark $	CRTC1	MAML2	$\checkmark $	$\checkmark $

### Real datasets

We tested Genion and LongGF on three real dateset: (i) MCF-7 breast cancer cell line sequenced using PacBio (accession: PRJNA277461), (ii) UHR (Universal Human Reference) RNA that is composed of ten different cell lines [39] sequenced by ONT GridION (accession: PRJNA639366), and (iii) 22Rv1 prostate cancer cell line sequenced in-house using two flowcells on an ONT MinION sequencer.

For the MCF-7 cell line, we found a set of 16 validated gene fusions released by PacBio Systems 2. Three out 16 gene fusions are validated experimentally using RT-PCR and PET-0. The remaining fusions are validated using orthogonal short-read sequencing data. Since short read validation does not account for chimeric artifacts, these calls cannot be considered with as much confidence. Actually Genion successfully discovered several of them but annotated them as random pairings (Table 3). We included them in this manuscript because they are the closest data available that can be considered as gold standard. For UHR and 22Rv1, we did not find any validated fusion.

**Table 3:** Genion and LongGF gene fusion calls on MCF-7 cell line data released by PacBio. These set of gene fusions are validated either experimentally (EXP) or by short reads sequencing (SRS). *#* Reads is the number of reads from the data release. Ranks are the order which fusion reported by the tools. Chimeras found by Genion, but identified as random pairing are given RP rank. FiN and ff-igf are the scores computed by genion

Gene1	Gene2	# Reads	Validation	LongGF	Genion			SV Overlap
				Rank	Rank	FiN	ff-igf	
BCAS4	BCAS3	1183	EXP	1	1	5.15582	8070.86	translocation
RPS6KB1	VMP1	349	EXP	2	2	0.25847	2046.15	deletion
SYTL2	PICALM	117	SRS	4	3	0.45365	857.222	✗
RPS6KB1	DIAPH3	101	SRS	5	4	0.31655	626.54	✗
SLC25A24	NBPF6	41	SRS	6	5	1.80952	472.95	inversion
PAPOLA	AK7	37	SRS	7	6	0.20536	369.71	✗
ESR1	CCDC170	24	SRS	10	8	0.52564	308.07	✗
TXLNG	SYAP1	27	EXP	12	9	0.11986	275.28	✗
TBL1XR1	RGS17	15	SRS	34	12	0.66667	153.69	translocation
MYO6	SENP6	14	SRS	35	13	0.10683	147.50	✗
POP1	MATN2	7	SRS		RP	0.15790	28.27	✗
MYH9	EIF3D	11	SRS	14	RP	0.04000	140.16	✗
FOXA1	TTC6	26	SRS			4.10526	303.47	inversion
RSBN1	AP4B1	7	SRS	19		0.01100	8.00	✗
ZNF217	SULF2	16	SRS		RP	0.00758	27.14	deletion
ARFGEF2	SULF2	23	SRS	15	RP	0.03104	113.28	inversion

**Fig. 3:**
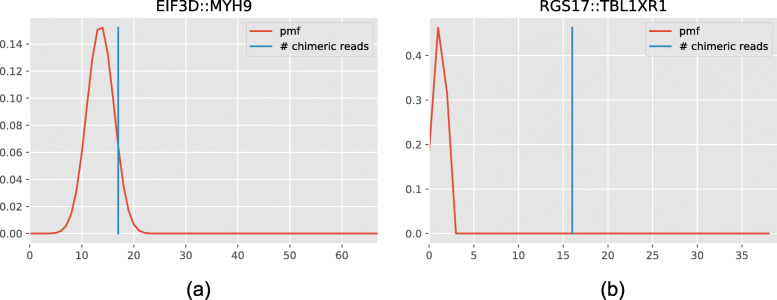
Probability mass functions (pmf) of **a** EIF3D:MYH9 and **b** RGS17:TBL1XR1 chimeras derived from the hypergeometric distribution. Red line shows the probability of getting number of random pairings for the candidate, blue line shows the number of chimeric reads found by Genion. *P*-value of the candidate is calculated by the area below the pmf after the blue line

In MCF-7, Genion called 22 gene fusions and 15 read-through events. LongGF called 297 gene fusions and 155 of them had at least 3 read support and we used those calls for a fair comparison. Genion and LongGF shared 17 gene fusion calls and 69 out of 155 LongGF calls were filtered by Genion as random pairings. As shown in Table 3, both LongGF and Genion successfully identified the three experimentally validated gene fusions (BCAS3:BCAS4, RPS6KB1:VMP1 and SYAP1:TXLNG). LongGF identified 10 out the 13 remaining validated gene fusions while Genion reported 8. From the five gene fusions that are not identified by Genion: FOXA1:TTC6 was filtered out during the chaining process as these two genes do overlap, POP1:MATN2, MYH9:EIF3D, ZNF217:SULF2, and ARFGEF2:SULF2 were filtered out as random pairings because they do not have significantly more read support than what is expected from a random pairing, and RSBN:APB1 has low read support. Probability mass functions used to determine if a chimera is random pairing are shown in Fig. 3 for EIF3D:MYH9 and RGS17:TBL1XR1 chimeras. It is important to note that the FOXA1:TTC6 fusion has good FiN and ff-igf scores and is supported by a genomic event nearby. Thus, we believe our overlapping gene filter might be too stringent. ZNF217 and SULF2 are observed in 39 and 104 other chimeric clusters, respectively, which supports this chimera being a false positive. LUMPY [40] reported a deletion of ∼5.8Mb between these two genes, however the power of short reads in calling a deletion of this magnitude is at best dubious without analyzing the depth of coverage in this region. Contrarily, the ARFGEF2:SULF2 fusion shows similar expression to its head ARFGEF2 gene and it is more likely to be a real gene fusion than other SULF2 fusion in the set ZNF217:SULF2. This may be a shortcoming of the FiN score which may thus leave room for improvement. RSBN1:AP4B1 had low support and Genion did not report it. Upon closer inspection we see that Genion reported RSBN1:AP4B1-AS1 where AP4B1-AS1 is the antisense RNA1 of AP4B1. This may be caused by our underlying mapper as our chaining prefers to build the chain on the AP4B1-AS1 rather than AP4B1.

Furthermore, we downloaded WGS data for the MCF-7 cell line and identified Structural Variations (SV) using LUMPY, a state-of-the-art short reads SV caller with a good balance of precision and recall. LUMPY identified 2,958 deletions and 67 inversions on this dataset. Gene fusions caused by genomic events should overlap with SVs, unlike read-through events. Furthermore, overlapping SVs should be compatible with the strands of the each gene in a fusion pair: where fusions involving genes on the same genomic strand should be associated to deletions, fusions involving genes on the inverse strands should be supported by inversions, and fusions involving genes on different chromosome should be supported by translocations. We manually investigated the SVs around these 16 gene fusions and identified 6 events to be compatible with our called gene fusions. It is interesting to note that 1 out of the 3 experimentally validated fusions (Table 3) did not overlap with any of the LUMPY calls suggesting that WGS short reads data might not always allow to detect SVs causing gene fusions, which is expected due to the shortcomings of SV detection from short reads, especially for large events.

As mentioned earlier, there is no ground truth information for the UHR cell line, thus we rely on information gathered from the literature. For the UHR cell line, both tools predicted the BCAS3:BCAS4 and VMP1:RPS6KB1 fusions, which are also observed as top calls in the MCF-7 cell line. This is expected as UHR contains cell lines derived from mammary gland carcinoma [39]. VMP1:RPS6KB1 was predicted as a read-through by Genion because of the low percentage of chimeric reads. LongGF also predicted the ARFGEF2:SULF2, SLC27A6:ADAMTS19 and MGAT5:IGLC7 fusions. ARFGEF2:SULF2 fusion was also called for MCF-7 cell line and SLC27A6:ADAMTS19 was previously identified in breast cancer with short reads [41]. Both of these chimeras were found by Genion and rejected due to their low support.

We tested both tools on the 22Rv1 cell line which is a prostate cancer cell line known for expressing different isoforms of the AR gene [42]. To the best of our knowledge, this cell line does not have any reported gene fusion and we think it is not enriched for gene fusions. Genion called only the ARHGAP15:GTDC1, HOXA5:HOXA6 and KISS1:GOLT1A gene fusions and 12 read-throughs, while LongGF called 89 gene fusions including ARHGAP15:GTDC1. The Depmap database [43] contains an inversion event on top of ARHGAP15 and GTDC1, which supports this fusion event, however to the best of our knowledge, this fusion has not been validated.

Finally, we tested both tools on NA12878, a germline dataset for which we do not expect any gene fusions. We used this dataset as a negative control. Genion did not report any gene fusions or read-through chimeras while LongGF called IGLV6-57:IGLC2, RPL29:MT-ND1 and ATP5IF1:RPS15A fusions.

**Resource Requirement.** In Table 4 we show the computational footprint of Genion and LongGF to process the MCF-7 Pacbio dataset. Genion has a lower peak memory that longGF, however, its running time is 3× longer than LongGF. Note that we also provide the time and memory required by minimap2 and deSALT as these two mappers are being utilized by LongGF and Genion, respectively.

**Table 4:** Time and memory used by Genion and LongGF during mapping and fusion calling steps on PacBio sequencing of MCF-7 breast cancer cell line

	Mapping			Fusion Calling		
	Threads #	Time (mm:ss)	Peak Memory (GB)	Threads #	Time (mm:ss)	Peak Memory (GB)
LongGF/minimap2	48	16:47	44.94	1	05:28	1.34
Genion/deSALT	48	24:07	37.25	1	14:36	0.85

## Discussion

In this work we present a computational tool that detects gene fusions on WTS long-read data. Many state-of-the-art methods rely on manual curation of the called fusions with a genome viewer (i.e. IGV). While this is a reliable method for filtering false positives, it requires a lot of manual work, and the decreasing cost of sequencing will likely increase the amount of datasets and required analysis. Thus, a gene fusion detection method that does not require human validation is essential. In this work, we present Genion, a tool that quantifies the quality of the called fusions and generate high quality calls with minimal manual filtering. In our experiments on simulated data Genion was able to find previously validated gene fusions and to differentiate read-through candidates from real gene fusions. While at the moment Genion uses deSALT, a mapper that only returns one optimal mapping, we designed the chaining algorithm with multi-mapped reads in mind, where the algorithm picks the best segments from many secondary mappings.

LongGF works by applying series of filters to the reads before clustering (binning) them. These filters can be summarized as filtering overlapping genes, overlapping alignments and distant alignments (on read). Instead of filtering individual reads, Genion applies filters to whole read clusters. While this approach is relatively slower than filtering individual reads, it gives greater filtering power and better information to analyse the filtered candidates. Similar to LongGF, Genion filters candidates based on the overlaps of the genes and alignments. However, Genion does not filter reads with long distance between alignments on the read. While distance between alignments is a common signature we observe on false positives, it still can be observed on real candidates in the presence of mapping errors and other genomic variants (i.e. insertions). A novelty of Genion is the statistical testing of the clusters in terms of number of fusion reads and normal reads from its member genes.

A possible future direction for this work is finding gene fusions on single cell long reads. This is a long-read sequencing method that uses 10X Genomics barcoded that is normally used in short reads. Finding gene fusions in such data can provide insight into heterogeneity in tumours.

Another avenue for further developments concern read-throughs. It is difficult to characterize read-through events using only transcriptome sequencing data. We suspect that read-through transcription may be related to chromatin accessibility of head and tail genes. Joint analysis with ATAC-seq might be able to provide insight on read-through transcription.

## Conclusions

In summary, Genion is an accurate gene fusion discovery tool that uses a combination of dynamic programming and statistical filtering. We believe it will be an effective tool for analyzing long transcriptomics sequencing data.

## Data Availability

Genion is implemented in C++ and is available at https://github.com/vpc-ccg/genion. Pacbio Iso-Seq sequencing of MCF-7 cell line can be accessed from PRJNA277461. WGS sequencing of MCF-7 cell line can be accessed from PRJNA523380. ONT Direct RNA sequencing of UHR can be accessed from PRJNA639366. In-house MinION direct cDNA sequencing of 22Rv1 cell line can be accessed from PRJNA726724.

## Abbreviations

DNA: Deoxyribonucleic acid
RNA: Ribonucleic acid
cDNA: Complementary DNA
TLRS: Transcriptomics Long-Read Sequencing
ff-igf: Fusion Frequency Inverse Gene Frequency
FiN: Fusion inverse Normal
FDR: False Discovery Rate
ARI: Adjusted Rand Index
TP: True positive
TN: True negative
FP: False positive
FN: False negative
SV: Structural Variation
ONT: Oxford Nanopore Technonologies
UHR: Universal Human Reference RNA

## Footnotes

1 We opened the following issue in their github on Jan 15, 2021: https://web.archive.org/web/20220205030953/https://github.com/SchulzLab/Aeron/issues/25

2 Accessed from: https://web.archive.org/web/20220204004810/https://github.com/PacificBiosciences/DevNet/wiki/IsoSeq-Human-MCF7-Transcriptome.
